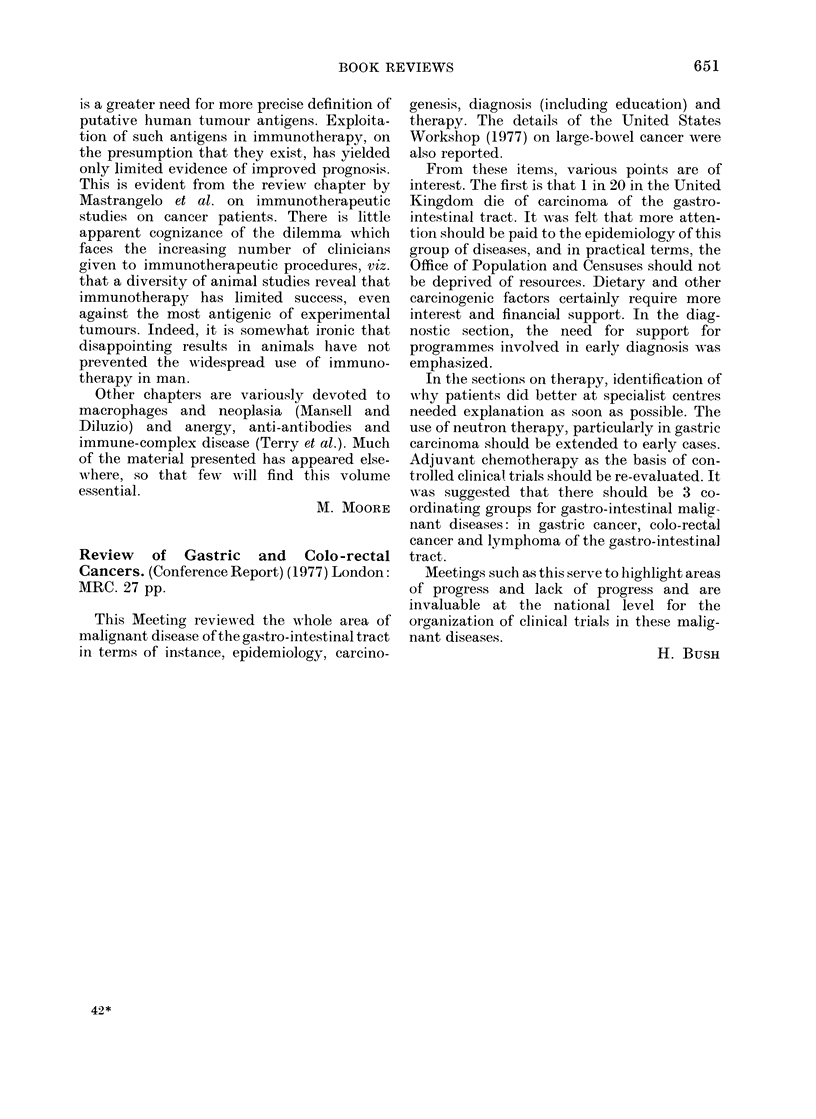# Review of Gastric and Colo-rectal Cancers

**Published:** 1978-04

**Authors:** H. Bush


**Review of Gastric and Colo-rectal Cancers.** (Conference Report) (1977) London: MRC. 27 pp.

This Meeting reviewed the whole area of malignant disease of the gastro-intestinal tract in terms of instance, epidemiology, carcinogenesis, diagnosis (including education) and therapy. The details of the United States Workshop (1977) on large-bowel cancer were also reported.

From these items, various points are of interest. The first is that 1 in 20 in the United Kingdom die of carcinoma of the gastro-intestinal tract. It was felt that more attention should be paid to the epidemiology of this group of diseases, and in practical terms, the Office of Population and Censuses should not be deprived of resources. Dietary and other carcinogenic factors certainly require more interest and financial support. In the diagnostic section, the need for support for programmes involved in early diagnosis was emphasized.

In the sections on therapy, identification of why patients did better at specialist centres needed explanation as soon as possible. The use of neutron therapy, particularly in gastric carcinoma should be extended to early cases. Adjuvant chemotherapy as the basis of controlled clinical trials should be re-evaluated. It was suggested that there should be 3 co-ordinating groups for gastro-intestinal malignant diseases: in gastric cancer, colo-rectal cancer and lymphoma of the gastro-intestinal tract.

Meetings such as this serve to highlight areas of progress and lack of progress and are invaluable at the national level for the organization of clinical trials in these malignant diseases.

H. BUSH